# Volume Management in Pulmonary Arterial Hypertension Patients: An Expert Pulmonary Hypertension Clinician Perspective

**DOI:** 10.1007/s41030-018-0052-z

**Published:** 2018-06-01

**Authors:** Lillian Hansen, Marsha Burks, Martha Kingman, Traci Stewart

**Affiliations:** 1 University of Arizona, Tucson, AZ USA; 2 University of Michigan, Ann Arbor, USA; 3 University of Texas, Southwestern Medical Center at Dallas, Dallas, TX USA; 4 Heart and Vascular Center, University of Iowa, Iowa, USA

**Keywords:** Pulmonary arterial hypertension, Volume management, Volume overload

## Abstract

Fluid volume management in patients with pulmonary arterial hypertension (PAH) is essential in preventing right ventricular failure. Volume overload may be caused by disease progression, indiscretion of dietary sodium and fluid intake, or medication side effects, and is a frequent complication in patients with PAH. Healthcare professionals (HCPs) who care for patients with PAH have a key role in monitoring, preventing, and managing volume overload. Volume management techniques in patients with PAH include managing diuretic use and electrolyte imbalances, and monitoring fluid retention that can occur from the use of endothelin receptor antagonists or calcium channel blockers. Healthcare providers can create volume management protocols as well as patient educational materials. Patients should be educated to self-monitor their daily weights, incorporate dietary restrictions, and recognize symptoms associated with volume overload. Tools to help HCPs with volume management in patients with PAH are provided in this article.

*Funding* Actelion Pharmaceuticals US, Inc.

## Introduction

Pulmonary arterial hypertension (PAH) is a progressive and fatal disease with complex hemodynamic and pathophysiological characteristics defined as a resting mean pulmonary artery pressure (mPAP) ≥ 25 mmHg, pulmonary capillary wedge pressure (PCWP) ≤ 15 mmHg, and pulmonary vascular resistance (PVR) > 3 Wood units as measured by right heart catheterization (RHC) [1]. In PAH, vasoconstriction of the pulmonary vascular bed occurs through endothelial and smooth muscle cell dysfunction, and in conjunction with thrombosis in situ and pulmonary artery wall remodeling, leads to increased afterload on the right ventricle (RV) [2]. The RV plays a pivotal role in maintaining pulmonary circulation as a low-pressure, high-volume system under normal circulation [3]. In PAH, the increased afterload in the pulmonary circulation leads to RV remodeling and ultimately failure through various mechanisms [4]. Initially, increased PVR results in RV dilation and RV diastolic and systolic dysfunction with decreased RV stroke volume [5]. Over time, diastolic ventricular interdependence between the RV and left ventricle (LV) leads to under-filling of the LV, resulting in reduced cardiac output, systemic hypotension, and subsequent release of antidiuretic hormone [3, 6]. Renal hypoperfusion and congestion occurs, which activates the renin–angiotensin–aldosterone system [4, 7]. In combination, these neurohormonal changes contribute to increased fluid retention, a hallmark sign of RHF [6].

In addition to fluid retention, clinical manifestations of RHF in patients with PAH include progressive dyspnea, elevated jugular venous pressure, and exercise intolerance [5]. Depending upon the severity of the RHF and fluid retention, outpatient management may be effective and preferred, however, in severe resistant cases, patients may require more aggressive inpatient management. Hospitalization for RHF is associated with increased mortality in patients with PAH and HCPs caring for patients with PAH play a pivotal role in the prevention and management of RHF and associated hospitalization [8]. To prevent RHF in patients with PAH, pressure and volume overload must be mitigated to decompress the RV and promote LV filling [5]. Patients are treated with pulmonary-specific vasodilators to reduce pressure overload [5]. Diuretics are the mainstay of treatment for volume overload in PAH and are effective in reducing right ventricular wall stress and tricuspid regurgitation [5].

While numerous publications exist for volume management in left heart failure, there is a paucity of literature on volume management in PAH and there are no published randomized controlled trials studying diuretic therapy in PAH. To address this lack of referenceable material, this article will focus on volume management in patients with PAH from an expert pulmonary hypertension clinician’s perspective. This article is based on previously conducted studies, clinical observations, and experiences of the authors and does not contain data from any new studies with human participants or animals.

## Diuretics

### Types of Diuretics

Several classes of diuretics are used in volume management in patients with PAH, all of which work by preventing reabsorption of sodium in the kidney and act on different areas of the nephron [9]. Loop diuretics, which act on the loop of Henle, are most commonly used because they are the most effective in inhibiting reabsorption of sodium [10]. Typically, patients are started on furosemide oral therapy for outpatient volume management. Patients will differ in their response to diuretics, and multiple strategies often need to be implemented to achieve diuresis. Failure to diurese can be managed by increasing the dose and/or frequency of the loop diuretic, changing to a different loop diuretic with higher bioavailability, or adding another form of diuretic (Tables 1, 2). For patients not responding to these strategies with oral diuretics, more advanced diuretic management options may include intravenous (IV) diuretics, paracentesis, ultrafiltration, or dialysis. A useful conversion for dosing is 0.5 mg bumetanide = 10 mg torsemide = 20 mg furosemide [9].

**Table 1:** Loop diuretics

Agent	Initial dose (mg)	Maximum dose (mg/day)	Approximate oral bioavailability (%)	Onset	Duration
Furosemide [45, 46]	20–80	600	60–64	Oral: 1 h IV: 5 min	Oral: 6–8 h IV: 2 h
Bumetanide [47, 48]	0.5–1	10	80–100	Oral: 30–60 min IV: 5 min	Oral: 4–6 h IV: 2–3 h
Torsemide [49]	10–20	200	80–100	Oral: 1 h IV: 10 min	Oral: 6–8 h IV: 6–8 h

**Table 2:** Thiazide-type diuretics

Agent	Route	Initial dose (mg)	Onset	Duration (h)
Hydrochlorothiazide [50]	Oral	12.5–25	2 h	6–12
Chlorothiazide [51]	Oral, IV	500	Oral: 2 h IV: 15 min	6–12
Chlorthalidone [52]	Oral	25–50	2.6 h	72
Metolazone [53]	Oral	2.5–5	1 h	> 24

Thiazides are a mainstay treatment for systemic hypertension and are also used in diuretic management (Table 2). Thiazide-type diuretics block sodium reabsorption in the distal tubule and can be added to loop diuretics to achieve diuretic synergy [10, 11]. Instructing a patient to take a thiazide 30 min prior to a loop diuretic can deliver more sodium to the loop of Henle to improve sodium excretion. Combination diuretic therapy can achieve up to double the amount of sodium excretion than loop alone; however risks include worsening kidney function, hypotension, and low serum sodium and potassium levels, all of which must be monitored closely [11]. A commonly used thiazide-like diuretic, metolazone, can be given as needed or on a scheduled basis to prevent or treat excess fluid accumulation and maintain euvolemia [12].

Aldosterone antagonists (AA) are a third type of diuretic used in the management of fluid overload in patients with PAH. In addition to contributing to fluid retention, aldosterone activation is associated with cardiac fibrosis and pulmonary vascular stiffening, thus theoretical benefits exist in blocking the effects of aldosterone in patients with PAH [13]. Limited data exist on the benefits of AA in PAH; however a current trial is underway investigating the use of spironolactone in PAH [14]. Spironolactone is a commonly used aldosterone antagonist diuretic, also referred to as a potassium-sparing diuretic, and often prescribed in combination with loop diuretics to achieve improved diuresis [5]. AA work in the collecting duct of the kidney and spare the loss of potassium during diuresis, which may help counter potassium loss induced by loop and thiazide diuretics. However, due to the risk of hyperkalemia, AA should only be initiated in patients with serum K levels < 5 and serum creatinine level < 2.0 in females and < 2.5 in males and require close laboratory follow-up for monitoring of serum potassium levels, as described below [15]. In addition, potassium supplementation may need to be reduced or discontinued when starting AA medications to avoid hyperkalemia.

## Risks Related to Diuretic Therapy

### Hypotension

Hypotension in patients with PAH may have multiple causes and can be impacted by diuretic therapy. Patients with PAH are often treated with medications that reduce systemic blood pressure and increase the risk of systemic hypotension, such as pulmonary vasodilators and calcium channel blockers for PAH and other antihypertensive agents for essential hypertension. In patients with severe PAH, systemic hypotension may be a sign of worsening RHF and end-stage disease [16, 17]. Preload reduction in PAH has been shown to improve LV filling and cardiac output (CO) by optimizing diastolic ventricular interdependence between the RV and LV [18]. Diuretics reduce preload through volume loss and may contribute to improving both LV CO and systemic blood pressure in patients with PAH. When attempting to manage volume overload in a patient with PAH, it is important to prevent worsening systemic hypotension, which can lead to an increased risk of dizziness or syncope. Caution should be taken to avoid overdiuresis and subsequent dehydration with diuretic therapy, and a reduction or discontinuation of systemic antihypertensive agents may be needed to allow for titration of diuretics without worsening hypotension.

### Electrolyte Imbalance

Common electrolyte imbalances seen with diuretic therapy include hypokalemia, hyperkalemia, hypomagnesemia, hyponatremia, and hyperuricemia [19]. Most electrolyte imbalances are not life-threatening, often asymptomatic, and can be easily managed with outpatient electrolyte replacement or diuretic adjustment [19]. However, some may require urgent management in the inpatient setting.

## Potential Fluid Retention Triggers

### Endothelin Receptor Antagonist (ERA) Therapy

Patients with PAH on pulmonary vasodilator therapy are at risk for developing edema, which can occur in patients who are treated with ERA medications [20, 21]. The etiology of edema from ERA therapy is not clearly defined; however, it is thought to be related to a combination of peripheral arterial vasodilation and blockage of endothelin receptors in the renal medulla [22]. Edema resulting from ERA therapy may be mild or severe, and thus treatments range from outpatient initiation of diuretics to inpatient diuretic management [17, 20]. Despite the possible development of edema, ERA therapy has shown clinical benefit for patients in pivotal trials [23–25]. When treating patients with ERA therapy, consider optimizing volume status prior to initiation and promptly adjust diuretics if edema develops or worsens. In addition, it is important to discern between edema as a medication side effect versus edema as a symptom of progressive RHF [21].

### Calcium Channel Blocker Therapy (CCB)

High-dose CCB therapy remains a treatment option for a small, select group of patients with PAH who demonstrate acute vasodilator responsiveness [1]. In addition, patients with PAH may be treated with CCB therapy for other comorbid conditions, including systemic hypertension, cardiac arrhythmias, and Raynaud’s phenomenon. Edema is a well-known side effect of CCB therapy, occurring more commonly in dihydropyridine CCBs (such as amlodipine, felodipine, and nifedipine) than in non-dihydropyridine CCBs (such as diltiazem and verapamil), and with higher-dose CCBs [26]. The mechanism of edema during CCB therapy is related to increased capillary hydrostatic pressure due to direct arterial precapillary vasodilation [27, 28]. This edema may not be sufficiently responsive to diuretic therapy, and instead may be better managed by CCB dose reduction, discontinuing CCB therapy, or changing to an alternative CCB [28].

### Non-steroidal Anti-inflammatory Drugs (NSAIDs)

NSAIDs are shown to affect renal function and can result in increased fluid and sodium retention, resulting in edema [29]. Therefore, routine use of NSAIDs in patients with PAH is typically cautioned against or contraindicated, depending upon the individual patient. When taken with loop diuretics, NSAIDs can reduce the diuretic response by up to 20%, and caution should be used when patients take NSAIDs with potassium-sparing diuretics due to an increased risk of hyperkalemia [29]. It is important to screen patients with PAH for NSAID use and discuss the relative risks and alternative pain management options. In general, similar caution should be applied to COX-2 inhibitors.

## Monitoring by the Healthcare Professional

### Outpatient Volume Management

**Fig. 1:**
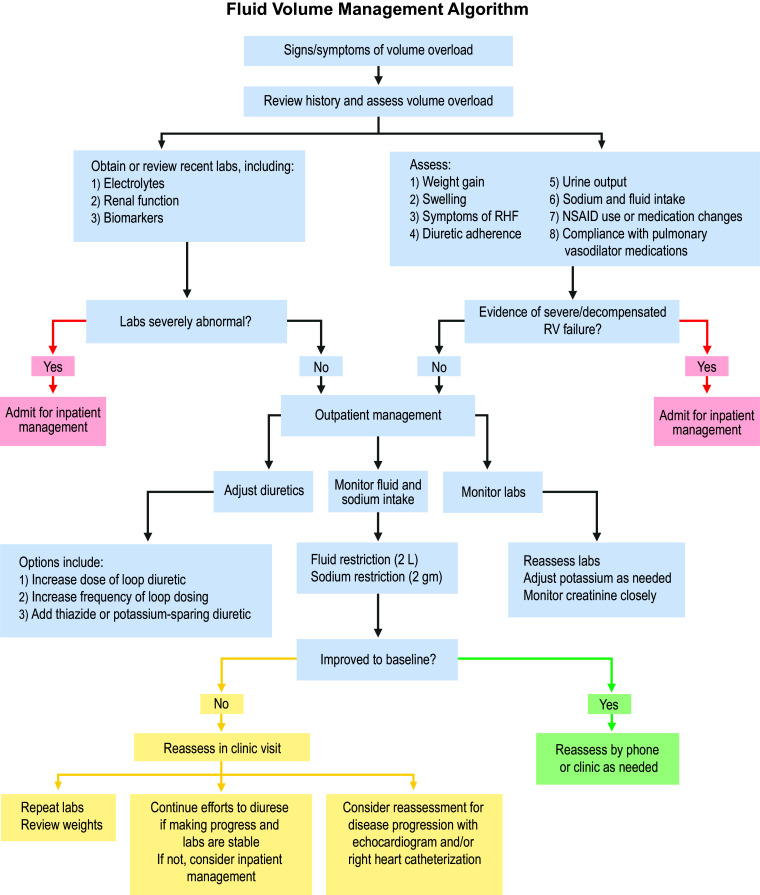
Healthcare professional protocol: volume management in patients with pulmonary arterial hypertension

**Fig. 2:**
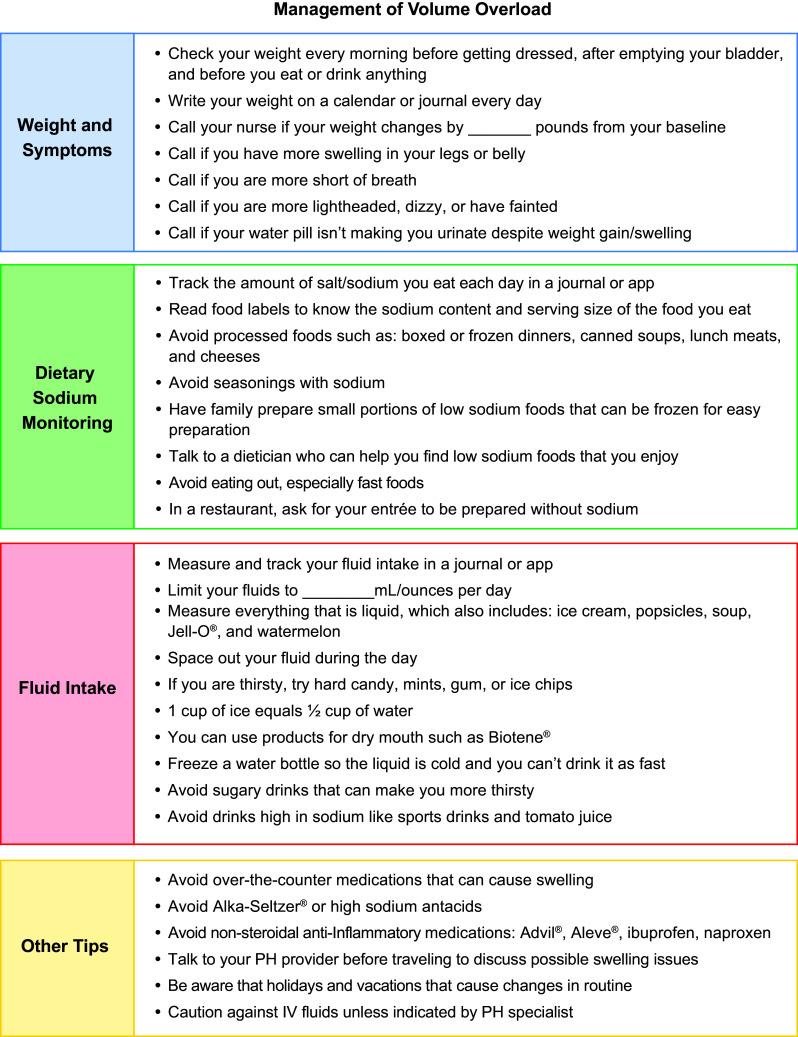
Handout for patients with pulmonary arterial hypertension outpatient management of volume overload

Volume overload is the most common cause of hospital admissions in patients with PAH, and hospitalization has been shown to correlate with a worse prognosis [8]. Thus, one of the most important outpatient HCP roles in the PAH program involves routine, close monitoring of fluid volume status. Volume overload issues that arise from dietary indiscretion or medication side effects may not require hospitalization if promptly identified and treated. Volume management involves not only the prescription of diuretics but also routine laboratory monitoring and extensive patient education around lifestyle modifications. In addition, in the case of progressive PAH or pulmonary vasodilator side effects, adjustments in pulmonary vasodilator therapy may be necessary. An example of an algorithm for outpatient management of volume overload in patients with PAH is found in Fig. 1 and suggested topics for patient education are found in Fig. 2.

### Diuretics

Aside from the patients with very mild PAH, most patients will require a routine maintenance dose of diuretics to manage RV volume overload [21]. Throughout the course of the disease with changes in volume status, patients may require adjustments to diuretic therapy. Increasing doses of diuretics may be needed with worsening RHF, dietary indiscretion, or medication side effects. Decreasing doses of diuretics may be required with worsening renal function due to volume depletion. Many patients will need a combination of diuretics, and in some cases, a change from furosemide to torsemide when absorption of furosemide is reduced due to bowel wall edema [30]. Other management strategies include use of a sliding scale diuretic regimen based on weight fluctuations [31]. For example, patients can be instructed to take metolazone when their daily weight exceeds a specified amount (Table 2). Additional potassium should be considered on days that metolazone is taken in order to prevent hypokalemia.

### Laboratory Monitoring

Patients treated with chronic diuretics will require surveillance laboratory monitoring to assess electrolytes and renal function. The frequency of laboratory monitoring depends on several factors including diuretic regimen, baseline renal function, and fluctuation in electrolytes. Typical laboratory tests include a basic metabolic panel as well as biomarkers such as N-terminal pro brain natriuretic peptide (NT-proBNP) or brain natriuretic peptide (BNP) [32]. Patients with potassium, sodium, or magnesium electrolyte abnormalities, and those with renal insufficiency, will need more frequent laboratory monitoring (Fig. 1). For loop and thiazide-like diuretics, consider checking electrolytes and renal function at baseline, within 1 week after initiation and dose adjustments, and monthly, quarterly, or biannually thereafter depending upon renal function [33].

Hypokalemia is common and can generally be prevented by administration of routine potassium chloride supplements and/or potassium-sparing diuretics (Table 3). Potassium-sparing diuretics are associated with an increased risk of hyperkalemia, especially in patients with chronic kidney disease, and thus need more frequent lab monitoring [19]. For example, consider checking potassium levels and renal function within 3 days of initiation of potassium-sparing diuretics and subsequently 1 week later, within 2 weeks after dose adjustments, and monthly until stability is obtained in potassium levels [15, 34]. Some programs may utilize protocol-driven potassium replacement to provide a more standardized approach that takes into account both the serum potassium level and glomerular filtration rate (Fig. 1) [35]. In addition, monitoring magnesium and avoiding low magnesium levels can help prevent hypokalemia.

**Table 3:** Potassium sparing diuretics

Agent	Initial dose	Maximum dose	Precautions
Spironolactone [54]	100 mg daily in single or divided dose	25–200 mg	Follow package insert for frequency of monitoring and dose adjustments of related to potassium and renal function
Eplerenone [55]	25 mg daily	50 mg daily	
Amiloride [56]	5 mg daily	Typical 10 mg daily but up to 20 mg daily	

When used for management of edema

Hyponatremia is associated with advanced RHF and markedly reduced survival in patients with PAH, independent of established hemodynamic, echocardiographic, and clinical markers of poor outcome, and thus should not be overlooked in the clinical assessment of patients with PAH [36]. Mild hyponatremia is addressed by restricting fluid intake, particularly free water [19]. For moderate hyponatremia, initiation of or increase in diuretic dose may be needed. The treatment for severe hyponatremia requires hospitalization and is beyond the scope of this paper. Increasing sodium intake should never be considered as a treatment option.

With rises in creatinine, the healthcare team will assess the need to make adjustments to the diuretic regimen. When changes are made, short-term follow-up with repeat laboratory analysis is recommended. For patients who require frequent laboratory monitoring, a standing order may be utilized. Generally, patients with chronic kidney disease will be more challenging to manage, requiring closer follow-up, more frequent laboratory testing, and possible collaboration with nephrology for diuretic management.

BNP is secreted by the cardiac myocytes and serves as a biomarker for acute and chronic disease severity in patients with PAH [37]. In a patient with PAH who has volume overload, BNP is likely to be elevated due to increased right ventricular strain and is expected to fall with appropriate diuresis and improved pulmonary vasodilation [38]. Higher baseline BNP is correlated with mortality in patients with PAH [32], and persistently elevated BNP, despite treatment, is predictive of reduced survival [38]. When caring for patients with PAH, routine monitoring of BNP or NT-proBNP is frequently performed and recommended by current guidelines [1]. For example, these biomarkers may be measured for a baseline at diagnosis, after treatment initiation, and routinely every 3–12 months for surveillance purposes [1].

## Patient Education for Self-Monitoring

Healthcare providers should educate patients to monitor daily weights and track sodium and fluid intake (Fig. 2). Patients will need to be trained to recognize symptoms associated with volume overload and promptly report such symptoms to their HCP for adjustments in diuretic therapy and to avoid hospitalization. When fluid retention is identified, it is necessary to obtain a detailed history to identify precipitating factors (Fig. 1).

Effective communication between HCPs and patients is necessary for outpatient volume management and reduction in hospital admissions [39]. Many HCPs rely on telephone communication or in-person clinic visits, however, newer methods exist for patient tracking and communication of symptoms. For example, numerous mobile phone apps are available for tracking of heart failure symptoms, vital signs, medications, and dietary intake, however, these are not specific to RHF in PAH and many of the apps are lacking in quality, content, and functionality [39]. Remote patient monitoring through telemedicine has shown variable clinical benefit for patients with heart failure and remains a topic of ongoing research [40]. While there is a lack of evidence and specific recommendations for the use of mobile phone apps and telemedicine in PAH, findings from the heart failure literature can be adopted for PAH patients and future studies would be helpful.

### Daily Weights

Patients should be taught to weigh themselves daily in the morning after emptying their bladder and before getting dressed. They should record these daily weights and be instructed to contact the PAH provider when their weight increases from their baseline. For example, patients are asked to report if their weight increases by 2–3 lbs overnight or by 5 lbs in 1 week. Patients should bring their weight log to each clinic visit for review by the PAH providers. While weight gain occurs frequently in patients with PAH, keep in mind that weight loss can also occur due to disease progression, early satiety, cardiac cachexia, volume depletion, or prostacyclin side effects, including nausea, vomiting, or diarrhea.

### Dietary Modifications

Sodium and fluid restriction help mitigate the clinical manifestations of RHF that often complicate PAH and may be an under-utilized strategy for managing volume overload. A low-sodium diet is essential in maintaining euvolemia in patients with PAH; most often, patients are placed on a 2 g per day sodium diet and need to be carefully instructed on how to accomplish this [23]. Teaching includes the process of properly reading food labels to determine the amount of sodium present. Healthcare providers can quickly and easily provide education around sodium and fluid restriction using printed supplemental materials [41]. Either the nurse or dietitian should also instruct patients on foods high in sodium, such as processed foods, canned foods, or meals prepared in restaurants (Fig. 2). As patients are beginning a low-sodium diet, it may be helpful to ask them to keep a strict written diet history until they learn the sodium content of the foods they are choosing. Patients should be instructed to bring the sodium log to clinic for review by the PH team or dietician.

Many PH specialists will place patients on a fluid-restriction regimen, often 2 L per day. When beginning a fluid restriction regimen, patients should carefully measure all fluids, including anything liquid at room temperature. Fluids, such as that in watermelon, should be minimized or factored into the fluid restriction (Fig. 2). Monitoring fluid intake can be accomplished in a variety of ways, including filling an empty 2 L bottle with water each morning and removing water from it whenever fluid is consumed in order to visually see how much fluid is remaining for the day.

## Outpatient Scenarios and Illustration of Fluid-Volume Management Algorithm

Limited published algorithms on fluid-volume management exist in the literature and are primarily for the care of patients with left heart failure [42]. While diversity exists among available algorithms in managing heart failure, they are similar to the goal of reducing symptoms of heart failure and preventing hospitalization with optimized outpatient management. The algorithm below provides general direction for HCPs in the management of fluid overload in patients with PAH with RHF, integrating concepts of volume management discussed above and can be adapted to fit the needs of the individual PAH practice.

### Patient Scenario #1—Non-adherence

Ms. X is a 55-year-old female patient with stable WHO Group I PAH associated with scleroderma treated with oral phosphodiesterase type 5 inhibitor (PDE-5i) monotherapy. Hemodynamics from the most RHC are as follows: mPAP 32 mmHg, PCWP 10 mmHg, CO 4.8 L/min, cardiac index (CI) 2.7 L/min/m^2^, and PVR 4.58 Wood Units. The patient calls the PAH clinic reporting weight gain of 4 lbs over the last 2 days and increased ankle edema. She reports eating a high-sodium meal at a restaurant the previous day. She is currently taking furosemide 40 mg daily and potassium chloride 20 mEq daily and reports good urinary output. Laboratory results from 2 weeks prior showed normal electrolytes and renal function, as follows: Na 138 mmol/L, K 4.6 mmol/L, BUN 19 mg/dL, creatinine 0.96 mg/dL, BNP 189 pg/mL, GFR > 60 mL/min. Home blood pressure was reported as 124/76 and heart rate was 84 bpm.

Per the algorithm (Fig. 1): The healthcare provider encouraged the patient to resume adherence to 2 g per day sodium and 2 L per day fluid restriction. Her furosemide dose was doubled to 80 mg daily and potassium chloride was increased by 50% to 30 mEq daily. The patient was instructed to continue the increased doses for 3 days and was called to reassess after the 3-day period. The patients reported a weight loss of 4 lbs back to baseline and resolution of swelling. She was instructed to resume her previous doses of furosemide and potassium chloride and report any further issues. The low-sodium diet was reiterated during every call.

### Patient Scenario #2—Progression of Disease

Mr. X is a 48-year-old male patient with WHO Group I idiopathic PAH and moderate RHF treated with combination infused prostacyclin therapy and oral ERA therapy. Hemodynamics from his most recent RHC are as follows: mean PAP 55 mmHg, PCWP 9 mmHg, CO 4.1 L/min, CI 2.16 L/min/m^2^, and PVR 11.22 Woods Units. The patient presents to the clinic with a weight gain of 12 lbs, 2 + pitting edema to the knees, and increased dyspnea. He admits to not weighing himself daily, but was at his baseline weight about 2 weeks ago. He reports maintaining a low-sodium diet and drinking approximately 2500 mL of fluid per day. He reports not voiding as well with his current diuretic regimen including furosemide dose of 80 mg daily, spironolactone 25 mg daily, and potassium chloride 20 mEq daily. Blood pressure was recorded as 112/64 with heart rate at 102 bpm. The most recent labwork was obtained 4 weeks ago.

Per the algorithm (Fig. 1): Repeat labs are obtained the day of the clinic visit, including a basic metabolic panel and BNP, as follows: Na 135 mmol/L, K 3.5 mmol/L, BUN 19 mg/dL creatinine 1.1 mg/dL, GFR > 60 mlLmin, and BNP 328 pg/mL. Furosemide is increased to 80 mg twice daily, potassium chloride increased to 20 mEq twice daily, and he is continued on spironolactone 25 mg daily. He is instructed to reduce fluid intake to 2000 mL per day. When called to reassess after 3 days, he reports a 2 lb weight loss, no change in swelling or dyspnea, and minimally increased urine output. At this point, metolazone 2.5 mg daily is added for 3 days, taken 30 min prior to the morning furosemide dose, with an additional 20 mEq potassium taken with each metolazone dose. The patient is reassessed at the clinic after 4 days and his weight has come down an additional 8 lbs, vital signs are stable, and swelling and dyspnea are much improved, but not back to baseline. The patient is instructed to discontinue the metolazone and continue furosemide 80 mg twice daily with potassium 20 mEq twice daily and spironolactone 25 mg daily. Education is reinforced on a low-sodium diet, 2000 mL fluid restriction, the importance of daily weighings, and keeping a weight journal. He is instructed to call the office if he experiences a 3 lb weight gain in 2 or 3 days or 5 lbs in a week. A week later, he reports being back to baseline weight, edema is resolved, and repeat labs show stable renal function and electrolytes, however, he continues to report increased dyspnea from baseline and BNP remains elevated. The patient undergoes repeat echocardiogram with findings concerning for disease progression and repeat RHC is planned for further assessment and potential augmentation of pulmonary vasodilator therapy.

## Inpatient Volume Management

Many patients with severe PAH and chronic RV failure may require hospitalization for management of acute decompensated right ventricular failure (ADRVF) [8]. Precipitating factors for ADRVF include disease progression, infection, anemia, increased sodium/fluid intake, acute pulmonary embolism, thyroid dysfunction, and arrhythmia [5]. Progressively elevated PVR leads to elevated right atrial pressure, which contributes to hepatic congestion and development of ascites. Renal congestion combined with reduced arterial renal perfusion also occurs, leading to diuretic resistance and worsening renal function secondary to prerenal azotemia. This phenomenon of worsening heart function contributing to worsening renal function is described as “cardiorenal syndrome” [43]. Reduced cardiac output and risk for atrial arrhythmias contributes to syncope and low systemic blood pressure [5].

When caring for a patient with ADRVF, ICU or telemetry admission is required depending upon the complexity of treatments and need for frequent monitoring [44]. Goals of care include improving volume status, organ perfusion, and oxygenation [5]. For volume management, IV loop diuretics, either continuous infusion or intermittent dosing, are instituted with frequent laboratory monitoring, typically once or twice daily. In addition, if not responding adequately to IV loop diuretics, patients often require intermittent IV, oral thiazides, and/or aldosterone antagonists. In severe cases, patients require low-dose dopamine or dobutamine to improve cardiac output and renal perfusion before effective diuresis is achieved [5]. In refractory situations, short-term hemodialysis or ultrafiltration may be considered. During diuresis, patients may have worsening renal function before improvements are seen and withholding diuretics in response to worsening renal function on admission is cautioned against, as this may perpetuate the cycle of cardiorenal syndrome. Nephrology consultation may be helpful for persistently abnormal renal function. Initiation or titration of pulmonary vasodilators is often required during ADRVF [5]. However, caution must be exercised to avoid systemic hypotension and worsening renal perfusion, so for this reason, inhaled nitric oxide or epoprostenol may be employed during the ICU admission [5].

Hospitalization for ADRVF is associated with increased mortality, both during admission and in the immediate post-discharge phase [8]. For patients with recurrent admissions for ADRVF and lack of response to additional medical management with maximum pulmonary vasodilators, referral for a lung transplant is often considered.

## Conclusions

Volume management is paramount in the care of patients with PAH. Patients with PAH are at risk for RHF, which is complicated by volume overload. Precipitating causes for volume overload in PAH include disease progression, dietary indiscretion, or medication side effects. Most patients with PAH require diuretic therapy for the management of volume overload and frequent adjustments in diuretic therapy are not uncommon. Healthcare providers caring for patients with PAH play a pivotal role in the monitoring, prevention, and management of volume overload (Fig. 1). Outpatient management requires extensive education to patients on topics of sodium and fluid restriction, daily weighings, and reportable conditions (Fig. 2). In addition, management may include the development and institution of diuretic protocols, patient educational materials, and frequent laboratory monitoring. A primary goal of outpatient volume management is prevention of hospital admission. Despite close monitoring of volume status, patients with progressive PAH may not be responsive to, or appropriate for, outpatient diuretic management and may require admission for interventions such as IV diuretics and possibly treatment for RV failure including IV vasodilators, inotropes, and vasopressors. The tools developed here are designed to assist the PAH HCP with educating patients and managing volume in patients with PAH.
